# Uncertainty-aware large language models for explainable disease diagnosis

**DOI:** 10.1038/s41746-025-02071-6

**Published:** 2025-11-18

**Authors:** Shuang Zhou, Jiashuo Wang, Zidu Xu, Song Wang, David Brauer, Lindsay Welton, Jacob Cogan, Yuen-Hei Chung, Lei Tian, Zaifu Zhan, Yu Hou, Mingquan Lin, Genevieve B. Melton, Rui Zhang

**Affiliations:** 1 Division of Computational Health Sciences, Department of Surgery, University of Minnesota, Minneapolis, MN USA; 2 Department of Computer Science, University of Chicago, Chicago, IL USA; 3 School of Nursing, Columbia University, New York, NY USA; 4 Department of Electrical and Computer Engineering, University of Virginia, Charlottesville, VA USA; 5 Division of Surgical Oncology, Department of Surgery, University of Minnesota, Minneapolis, MN USA; 6 Center for Learning Health System Sciences, University of Minnesota, Minneapolis, MN USA; 7 Department of Medicine, Division of Hematology, Oncology and Transplantation, University of Minnesota, Minneapolis, MN USA; 8 Division of Cardiac Electrophysiology, University of California San Francisco, San Francisco, CA USA; 9 Department of Biochemistry and Molecular Biology, Mayo Clinic, Rochester, MN USA; 10 Department of Electrical and Computer Engineering, University of Minnesota, Minneapolis, MN USA; 11 Institute for Health Informatics and Division of Colon and Rectal Surgery, Department of Surgery, University of Minnesota, Minneapolis, MN USA

**Keywords:** Computational biology and bioinformatics, Diseases, Health care, Mathematics and computing, Medical research

## Abstract

Explainable disease diagnosis, which leverages patient information (e.g., symptoms) and computational models to generate probable diagnoses and reasoning, holds strong clinical promise. Yet, when clinical notes lack sufficient evidence for a definitive diagnosis, such as the absence of definitive symptoms, diagnostic uncertainty commonly arises, increasing the risk of misdiagnosis. Despite its importance, the explicit identification and explanation of diagnostic uncertainty remain under-explored in artificial intelligence-driven systems. To fill this gap, we introduce ConfiDx, an uncertainty-aware large language model fine-tuned with diagnostic criteria. We formalized the task of uncertainty-aware diagnosis and curated richly annotated datasets that reflect varying degrees of diagnostic ambiguity. Evaluating on real-world datasets demonstrated that ConfiDx excelled in identifying diagnostic uncertainties, achieving superior diagnostic performance, and generating trustworthy explanations for diagnoses and uncertainties. Moreover, ConfiDx-assisted experts outperformed standalone experts by 10.7% in uncertainty recognition and 26% in uncertainty explanation, underscoring its substantial potential to improve clinical decision-making.

## Introduction

Automatic disease diagnosis involves using computational models to predict the most likely diagnosis given observation data from patients (e.g., signs, symptoms, laboratory values). In real-world scenarios, merely providing diagnostic predictions often lacks trustworthiness due to the black-box nature of these models^1^. Some key features, such as diagnostic explanations and adherence to clinical guidelines, are essential to enhance trust and accuracy^2^. Specifically, diagnostic explanations allow clinicians to assess the model’s reasoning^3^, improving trust, while adherence to guidelines ensures standardization and reliability^4^. Incorporating these key elements into diagnostic systems fosters greater trust and safety in clinical applications.

In practice, diagnostic uncertainty usually arises in clinical decision-making^5,6^. This is especially common among primary care or ICU patients presenting with symptom complaints whose clinical notes and other data lack sufficient evidence, such as definitive symptoms or conclusive laboratory findings, thereby posing challenges for reliable diagnoses^7^. A cross-sectional survey of 32 primary care clinics reported that 13.6% of patients’ consultations in primary care in the United States had missing clinical information^7^. Similarly, a retrospective study reported that nearly 48% of psychiatric emergency department cases lack sufficient physical examinations^8^. In such cases, identifying diagnostic uncertainty enables clinicians to address gaps in evidence, minimizing misdiagnoses and adverse outcomes^7,9–11^. For example, an ICU patient presenting with chest pain, shortness of breath, and fatigue, but without definitive coronary angiography findings, may face diagnostic uncertainty between acute myocardial infarction^12^ and Takotsubo cardiomyopathy^13^. Although these conditions share similar presentations, they differ markedly in etiology, severity, treatment, and outcomes^14^. In such cases, automatically identifying diagnostic uncertainty and explaining it can help prevent misdiagnosis, yielding considerable value.

Despite the development of advanced diagnostic systems^15,16^, including those based on large language models (LLMs), current efforts struggle to recognize and explain diagnostic uncertainty^2,17,18^. Specifically, most systems rely on supervised deep learning trained on extensive labeled datasets^15,16,19^. While effective at distinguishing between diseases, these systems face challenges in incorporating learned medical knowledge, such as clinical guidelines, to assess the sufficiency of patient information or generate factual explanations^20,21^. Recently, LLM-based diagnostic systems have shown exceptional promise in clinical decision-making^17,22,23^ by leveraging their generative capabilities and extensive medical knowledge to produce comprehensive diagnostic explanations^24^. These features position LLMs as valuable tools for recognizing and explaining diagnostic uncertainty. Pioneering studies^5,25^ have begun exploring diagnostic uncertainty estimation. For example, Savage et al.^5^ applied three commonly used confidence estimation methods^26^: confidence elicitation, token-level probability estimation, and self-consistency agreement to estimate the uncertainty degree of GPT^27^ and LLaMA^28^ models for disease diagnosis. However, these efforts face two critical limitations. First, LLM confidence often misaligns with factual accuracy^29^, as LLMs may exhibit overconfidence in erroneous diagnoses due to flawed internal knowledge^30^. Second, these approaches^5,25^ fail to provide narrative explanations for diagnostic uncertainty and the diagnoses, limiting their applicability in clinical practice.

Building an LLM-based diagnostic system that can effectively recognize and explain diagnostic uncertainty is challenging. In practice, clinicians are trained to adhere to diagnostic criteria^31^, identifying uncertainties when criteria are unmet and seeking additional evidence to resolve them^32^. This adherence reflects a specialized human preference in medical decision-making^33^. While LLMs, including those tailored for medical use^34^, are pre-trained on extensive biomedical corpora and fine-tuned for various tasks^35,36^, they are generally not aligned with this professional preference^37,38^. Therefore, aligning LLMs to rigorously follow diagnostic criteria and recognize diagnostic uncertainty remains under-explored.

This study aimed to address these gaps by developing uncertainty-aware LLMs for explainable disease diagnosis. Our contributions are threefold. First, we formally defined the task of uncertainty-aware diagnosis, enabling the recognition of uncertain cases alongside corresponding explanations. Second, we curated datasets with nuanced annotations to evaluate the trustworthiness of diagnostic models. Third, we proposed a tailored fine-tuning approach that integrated diagnostic criteria into the training process, aligning LLMs to rigorously adhere to these criteria for predictions. Building on open-source LLMs, we developed ConfiDx, which are customized diagnostic models to deliver accurate diagnoses while identifying and explaining diagnostic uncertainties. Experiments on the curated dataset demonstrated that ConfiDx outperformed off-the-shelf LLMs in diagnostic accuracy, enhanced recognition of diagnostic uncertainty, and provided reliable, comprehensive explanations. Furthermore, in expert-AI collaboration settings, ConfiDx-assisted experts outperformed independent experts by 10.7% in uncertainty recognition and 26% in uncertainty explanation. To the best of our knowledge, this is the first study to tackle uncertainty-aware diagnosis. Our work significantly advances the trustworthiness of LLM-based diagnostic models, securing reliable and explainable clinical decision-making.

## Results

### Dataset creation and evaluation framework

**Fig. 1:**
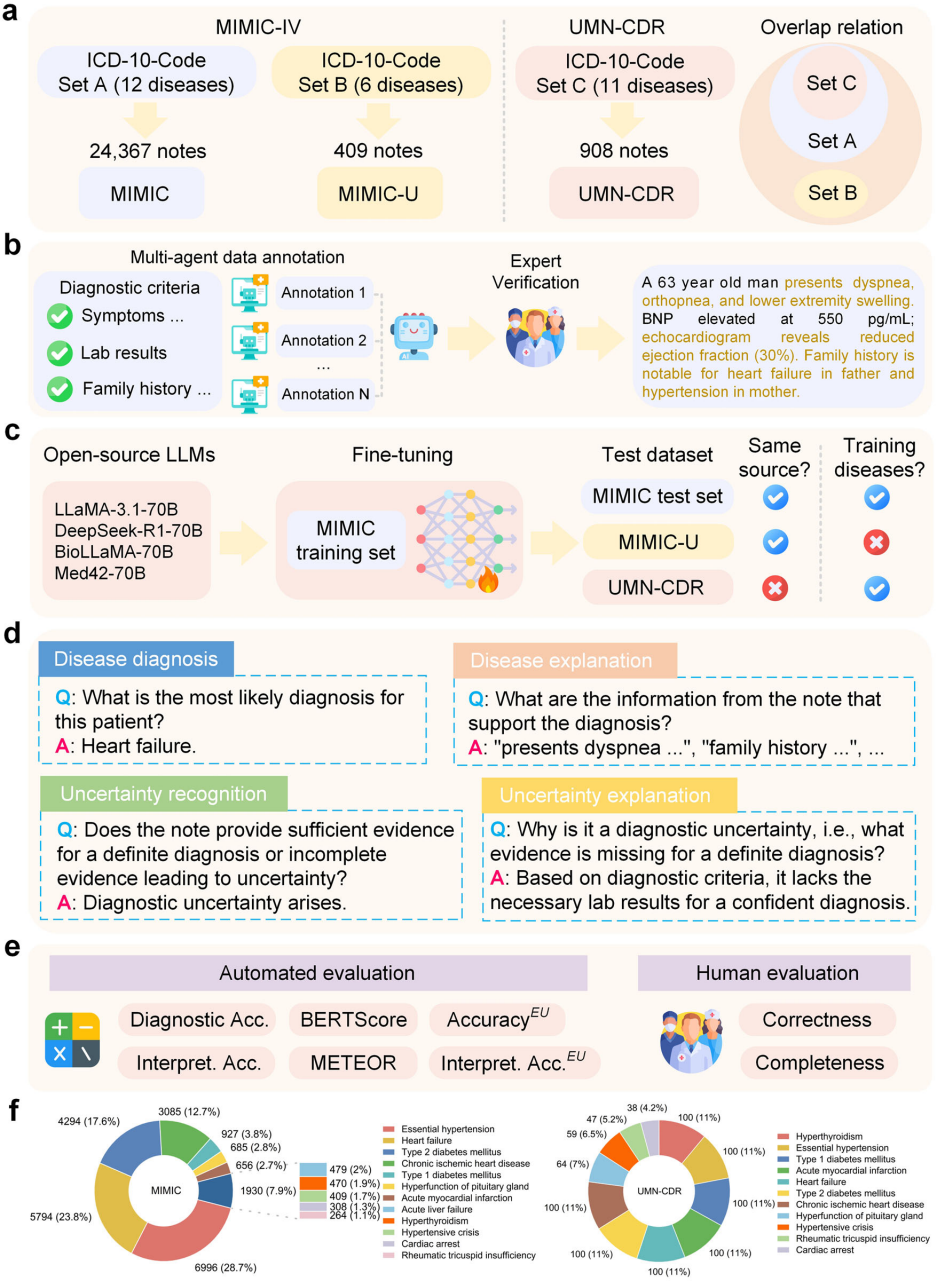
Overview of dataset creation, annotation, and evaluation framework. **a** To evaluate LLMs for disease diagnosis in realistic scenarios, we constructed three datasets using real-world clinical notes sourced from the MIMIC-IV and UMN-CDR databases. The disease types in the MIMIC dataset encompass those in the UMN-CDR dataset, whereas the MIMIC-U dataset features a distinct set of disease types. **b** Diagnostic explanations supporting the diagnoses were annotated on clinical notes based on manually curated diagnostic criteria. A multi-agent framework was developed for annotation, with subsequent verification by medical experts. **c** We fine-tuned open-source LLMs on the training data from the MIMIC dataset and assessed the performance on the MIMIC test set, MIMIC-U, and UMN-CDR datasets. Notably, MIMIC-U evaluates the model robustness on unseen diseases (i.e., absence from the training data), while UMN-CDR tests model generalizability across institutions. **d** Given that the target problem involves diverse prediction types (see “Definition formulation” in the Methods), we divided the problem into four manageable subtasks for performance evaluation. **e** Performance assessment involves various automated metrics complemented by human evaluation to ensure reliability. **f** Data composition of the constructed MIMIC and UMN-CDR datasets. Icons adapted from flaticon.com, used under royalty-free license.

We constructed datasets using clinical notes from the MIMIC-IV^39^ dataset and the University of Minnesota Clinical Data Repository (UMN-CDR) to develop and evaluate the proposed diagnostic models based on open-source LLMs (e.g., LLaMA). To assess model robustness on unseen disease types (i.e., absence from the training data), we created an independent test set, MIMIC-U, by holding out certain disease types from MIMIC-IV, leaving the rest as MIMIC. Additionally, we also collected publicly available case reports^40^ from PubMed Central (PMC) and The New England Journal of Medicine (NEJM) portal to assess large-scale commercial LLMs, such as GPT-4o and Gemini^41^, which generally cannot process privacy-sensitive clinical notes. We focused on three clinical specialties: endocrinology, cardiology, and hepatology, due to their significant impact on mortality in the U.S.^42^. Dataset construction details are in Fig. 1 and the “Datasets” section of the Methods, with statistics in Table 1. Data composition for MIMIC, UMN-CDR, and MIMIC-U is shown in Fig. 1f and Supplementary Note 1.

**Table 1:** Data statistics of the constructed MIMIC, MIMIC-U, and UMN-CDR datasets

Statistic	MIMIC	MIMIC-U	UMN-CDR
Total note number	24,367	409	908
Notes number in the training set	17,057	–	–
Notes number in the validation set	2436	–	–
Notes number in the test set	4874	409	908
Number of disease types	12	6	11
Total number of uncertainty cases	12,183	204	454
Mean note length (words)	1640	1686	955
Standard deviation of note length (words)	489	482	343
Mean number of explanations per note	3.15	3.71	3.36
Standard deviation of explanation number per note	0.83	1.23	0.89

In this study, we adopted four widely used open-source LLMs with 70 billion parameters (see “ConfiDx” section of the Methods). Given the complexity of uncertainty-aware disease diagnosis, which involves diverse prediction types (see “Definition formulation” in the Methods), we divided the problem into four manageable subtasks: disease diagnosis, diagnostic explanation, uncertainty recognition, and uncertainty explanation. We evaluated off-the-shelf and fine-tuned LLMs (ConfiDx) on these tasks, analyzing model robustness on hold-out disease types, generalizability across institutions, and key factors influencing performance.

### Uncertainty-aware diagnostic performance

**Fig. 2:**
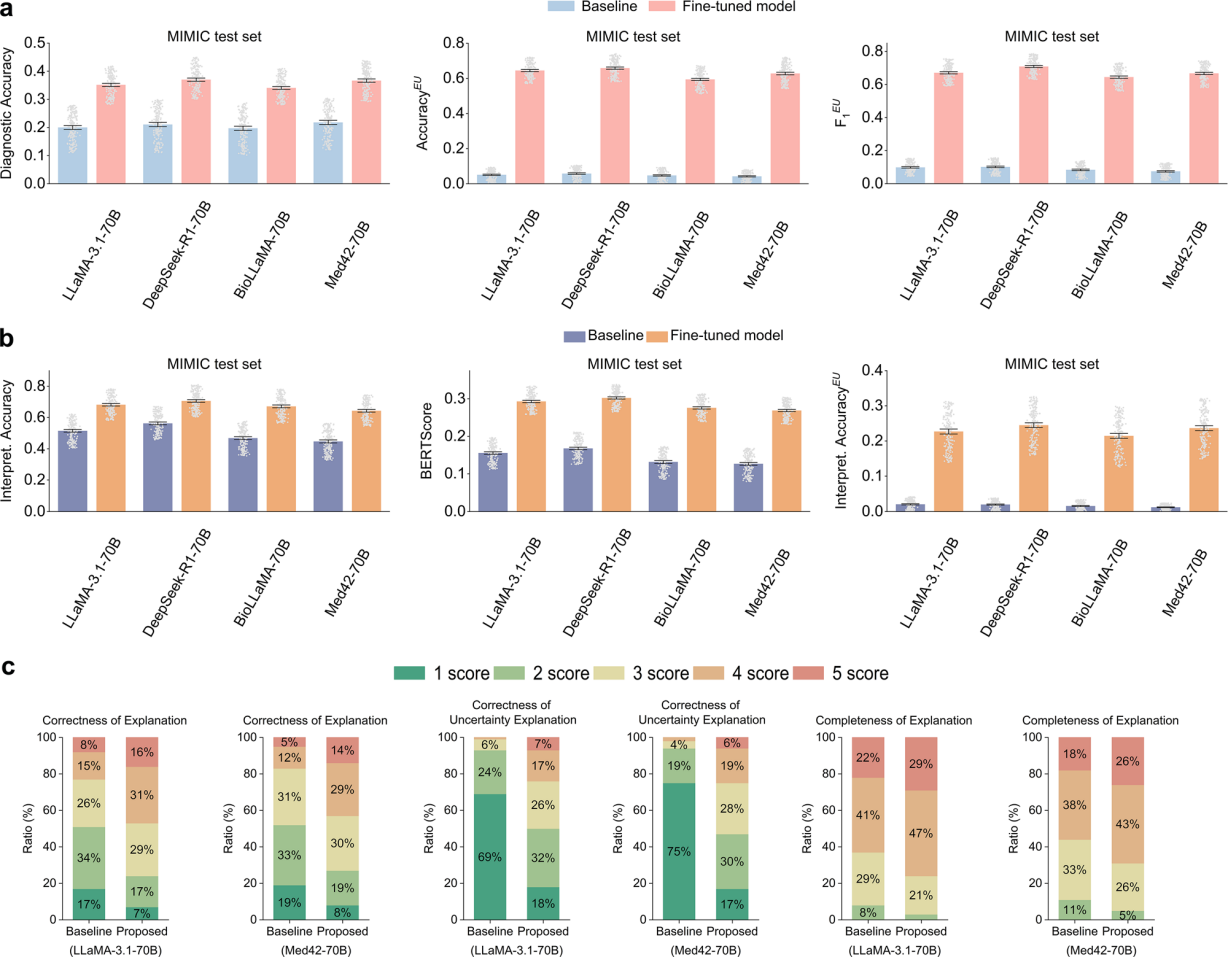
Performance of disease diagnosis and diagnostic explanation on the test set of the MIMIC dataset. **a** Performance comparison of baselines and the fine-tuned counterparts on disease diagnosis (measured by Diagnostic Accuracy) and diagnostic uncertainty recognition (measured by ${{Accuracy}}^{{EU}}$ and ${F}_{1}^{{EU}}$). Error bars represent the 95% CI of the mean, calculated via bootstrapping. **b** Automatic evaluation of explanations for disease diagnosis (using metrics such as Interpret. Accuracy and BERTScore) and diagnostic uncertainty (using Interpret. ${{Accuracy}}^{{EU}}$). Error bars represent the 95% CI of the mean, calculated via bootstrapping. **c** Results of manual evaluation of explanations, assessing the correctness and completeness of diagnostic explanations and the correctness of explanations for diagnostic uncertainty. Each metric was rated on a 5-point scale, where 1 represents the lowest and 5 is the highest score. Ratios below 3% are not highlighted in the figure.

The performance of disease diagnosis and diagnostic uncertainty recognition was evaluated on the MIMIC test set. The experimental prompts are detailed in Supplementary Note 2. As shown in Fig. 2a, the diagnostic accuracy of the off-the-shelf LLMs ranged from 0.197 (95% confidence interval (CI): 0.190–0.205) for BioLLaMA-70B to 0.218 (95% CI: 0.210–0.225) for Med42-70B. Fine-tuned models achieved accuracy improvements exceeding 68.3%, with all differences statistically significant (*p* < 0.001). For uncertainty recognition, the highest ${{Accuracy}}^{{EU}}$ and ${F}_{1}^{{EU}}$ scores among off-the-shelf LLMs were achieved by DeepSeek-R1-70B at 0.057 (95% CI: 0.054–0.062) and 0.102 (95% CI: 0.097–0.106), respectively. Remarkably, ConfiDx demonstrated substantial improvements, achieving ${{Accuracy}}^{{EU}}$ scores from 0.594 (95% CI: 0.587–0.600) for BioLLaMA-70B to 0.658 (95% CI: 0.652–0.665) for DeepSeek-R1-70B and achieving ${F}_{1}^{{EU}}$ scores from 0.644 (95% CI: 0.638–0.651) to 0.709 (95% CI: 0.702–0.715).

### Explanation performance

We evaluated the performance of the diagnosis and uncertainty explanation on the MIMIC test set, with prompts detailed in Supplementary Note 3. Partial results are presented in Fig. 2b, while additional metrics, including METEOR and SentenceBert, are provided in Supplementary Note 4. The interpretation accuracy of off-the-shelf LLMs ranged from 0.446 (95% CI: 0.437–0.456) for Med42-70B to 0.563 (95% CI: 0.554–0.571) for DeepSeek-R1-70B. Fine-tuning substantially improved performance, with gains ranging from 25.3% for DeepSeek-R1-70B to 43.8% for Med42-70B. For evaluation metrics such as BERTScore, SentenceBert, and METEOR, fine-tuned LLMs outperformed their off-the-shelf counterparts by an average of 96.7%, 63.4%, and 119.4%, respectively. Moreover, off-the-shelf LLMs demonstrated limited performance in uncertainty explanation, with an average performance of 0.017. In contrast, fine-tuned LLMs achieved superior accuracy, ranging from 0.214 (95% CI: 0.207–0.221) for BioLLaMA-70B to 0.245 (95% CI: 0.238–0.252) for DeepSeek-R1-70B, with performance improvements exceeding 0.2. All performance enhancements were statistically significant (*p* < 0.001).

Clinicians manually assessed explanations for diagnosis and uncertainty in terms of correctness^43^ and completeness^44^, as detailed in “Manual evaluation”. Figure 2c compares the performance of LLaMA-3.1-70B and Med42-70B. For diagnostic explanations, the correctness scores of off-the-shelf models predominantly ranged from 2 to 3, while fine-tuned models scored between 3 and 4. Regarding diagnostic uncertainty explanations, the two baselines received 69 and 75 scores of 1, while their fine-tuned counterparts achieved 50 and 53 scores above 2. In terms of completeness, the off-the-shelf Med42-70B achieved 38 scores of 4 and 18 scores of 5, while its fine-tuned counterpart achieved a superior explanation with 43 scores of 4 and 26 scores of 5.

### Robustness evaluation

**Fig. 3:**
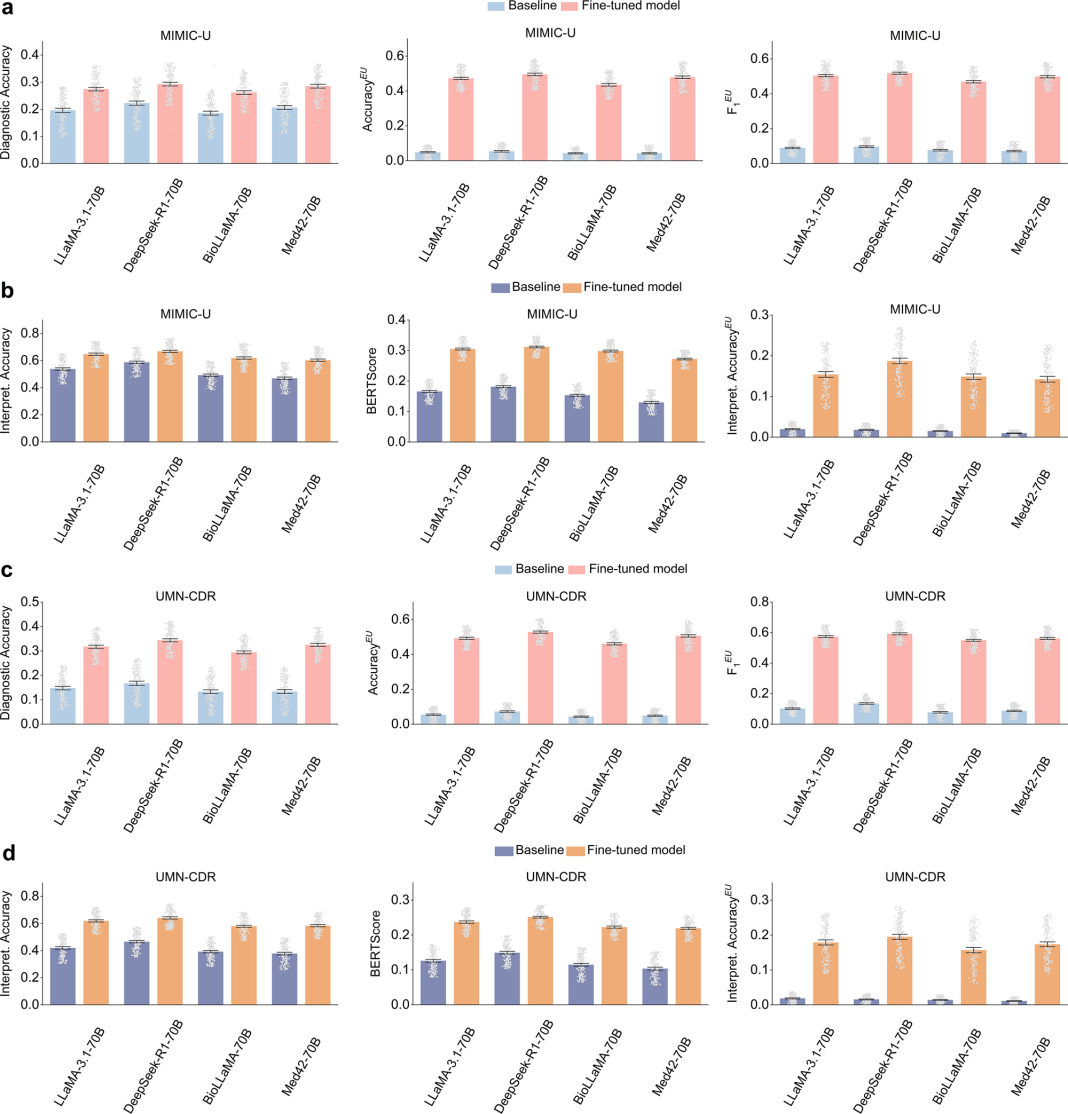
Robustness and generalizability evaluation performance on the MIMIC-U dataset with hold-out disease types and external UMN-CDR dataset, respectively. **a** Robustness performance on disease diagnosis (measured by Diagnostic Accuracy) and diagnostic uncertainty recognition (measured by ${{Accuracy}}^{{EU}}$ and ${F}_{1}^{{EU}}$). **b** Automatic evaluation of explanations for diagnoses (using metrics such as Interpret. Accuracy and BERTScore) and for diagnostic uncertainty (using Interpret. ${{Accuracy}}^{{EU}}$) on the MIMIC-U dataset. **c** Generalizability performance on disease diagnosis (measured by Diagnostic Accuracy) and diagnostic uncertainty recognition (measured by ${{Accuracy}}^{{EU}}$ and ${F}_{1}^{{EU}}$). **d** Automatic evaluation of explanations for diagnoses (using metrics such as Interpret. Accuracy and BERTScore) and for diagnostic uncertainty (using Interpret. ${{Accuracy}}^{{EU}}$) on the UMN-CDR dataset. Error bars represent the 95% CI of the mean, calculated via bootstrapping.

We further evaluated model robustness using the holdout MIMIC-U dataset, which includes diseases that ConfiDx did not see from the training data. Figure 3a presents the results for disease diagnosis and diagnostic uncertainty recognition. The diagnostic accuracy of ConfiDx ranged from 0.263 (95% CI: 0.257–0.270) for BioLLaMA-70B to 0.294 (95% CI: 0.287–0.300) for DeepSeek-R1-70B, representing performance improvements of 38.9%, 28.4%, 41.8%, and 39.3%, respectively, compared to their off-the-shelf counterparts. For diagnostic uncertainty recognition, fine-tuned models obtained an average ${{Accuracy}}^{{EU}}$ of 0.471 and an ${F}_{1}^{{EU}}$ of 0.497, marking substantial advancements over baseline performance of 0.046 on ${{Accuracy}}^{{EU}}$ and 0.083 on ${F}_{1}^{{EU}}$ (*p* < 0.001). The performance of the diagnostic explanation was also analyzed, as shown in Fig. 3b and Supplementary Note 5. Fine-tuned LLMs exhibited substantial improvements across all the explanation metrics, enhancing BERTScore by 60% and 49.2%, 56.2%, and 75.2%, respectively. Similarly, explanations for diagnostic uncertainty showed significant improvements, with an average score of 0.142. These ranged from 0.142 (95% CI: 0.135–0.149) for Med42-70B to 0.187 (95% CI: 0.180–0.194) for DeepSeek-R1-70B. These results demonstrate the ability of fine-tuned LLMs to perform effectively on the holdout MIMIC-U dataset.

### Generalizability evaluation

The model generalizability was evaluated through cross-institute validation using the UMN-CDR dataset. Figure 3c summarizes the results for disease diagnosis and diagnostic uncertainty recognition. Fine-tuned LLMs demonstrated significant improvements in diagnostic accuracy, with average increases of 0.169, 0.176, 0.162, and 0.186 across the four LLMs, respectively. For diagnostic uncertainty recognition, ConfiDx achieved an average ${{Accuracy}}^{{EU}}$ of 0.497 and ${F}_{1}^{{EU}}$ of 0.569, indicating substantial enhancements over their non-fine-tuned counterparts (*p* < 0.001). The explanation results are detailed in Fig. 3d and Supplementary Note 6, where ConfiDx consistently showed notable gains over the baselines across all explanation metrics (*p* < 0.001). For instance, fine-tuned LLaMA and DeepSeek improved BERTScore by 88.1% and 68.5%, respectively, and enhanced SentenceBert scores by 68.9% and 72.2%. Similarly, the performance of the diagnostic uncertainty explanation was significantly improved with an average of 0.162. The performance comparison shows that fine-tuned LLMs offer superior generalizability on the external dataset compared to their off-the-shelf counterparts.

### Comparison with large-scale LLMs

**Fig. 4:**
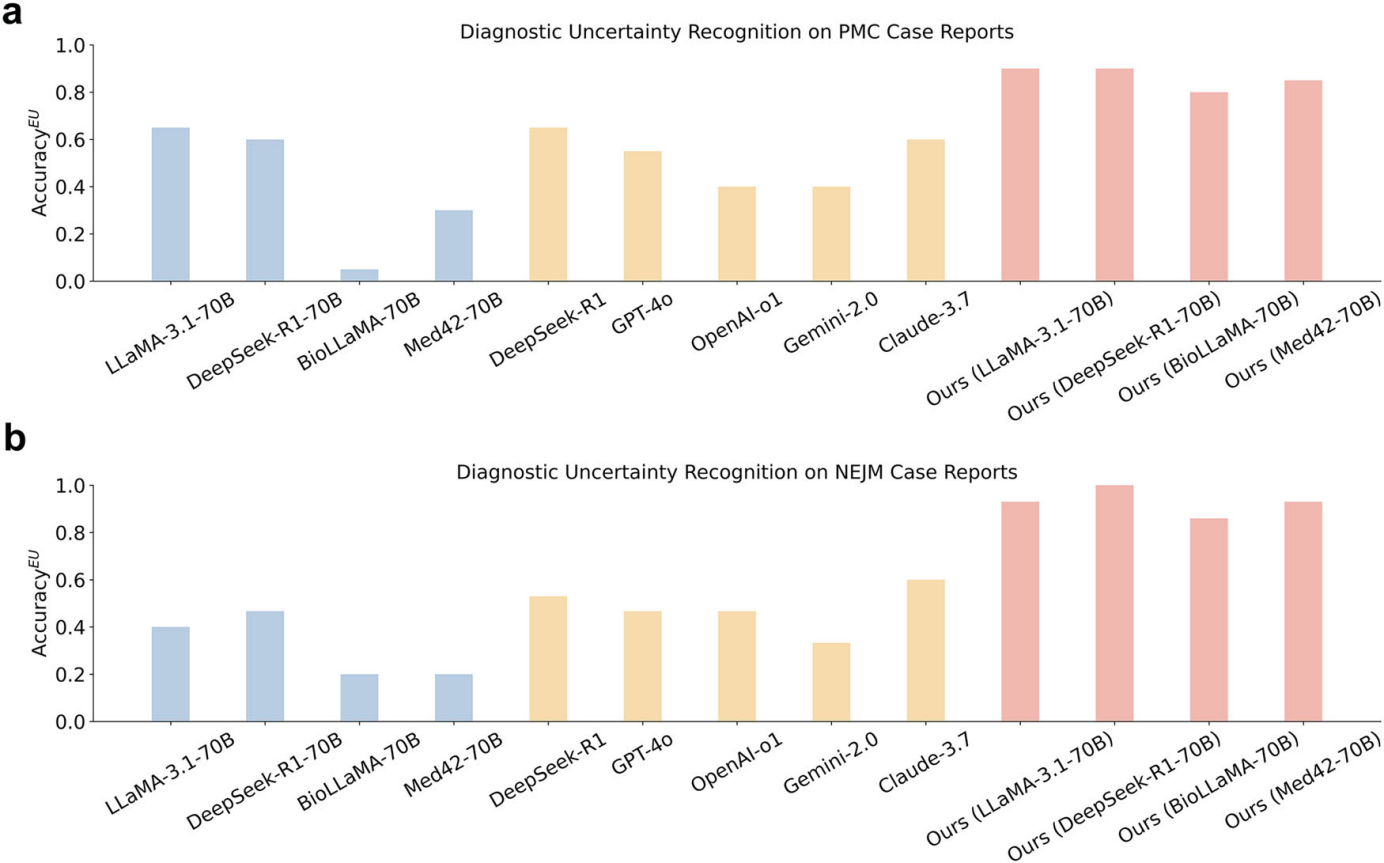
Performance of commercial LLMs in recognizing diagnostic uncertainty. **a** Comparison with large-scale LLMs on PMC case reports. **b** Comparison with large-scale LLMs on NEJM case reports. The blue, yellow, and red color denotes baselines, large-scale LLMs (i.e., with hundreds of billions of parameters), and our fine-tuned LLMs with 70 billion parameters, respectively. Our models consistently outperformed all the large-scale models.

We evaluated the performance of large-scale commercial LLMs, comprising hundreds of billions of parameters, in recognizing diagnostic uncertainty. Their results were compared with ConfiDx on the PMC case reports. The analysis included five widely used LLMs, i.e., GPT-4o, OpenAI-o1, Gemini-2.0^41^, Claude-3.7, and DeepSeek-R1^45^. As illustrated in Fig. 4a, the accuracy of large-scale LLMs ranged from 0.45 for OpenAI-o1 and Gemini-2.0 to 0.65 for DeepSeek-R1, with an average performance of 0.52, surpassing the average of 0.40 achieved by off-the-shelf LLMs with 70 billion parameters. For instance, DeepSeek-R1 failed to capture diagnostic uncertainty in a PMC case of primary biliary cholangitis, where the processed note did not meet the diagnostic criterion and, with lower likelihood, could also indicate autoimmune hepatitis. Likewise, Claude failed to recognize diagnostic uncertainty in an NEJM case of cardiac arrest, where the processed note did not satisfy the diagnostic criterion and could alternatively indicate syncope. In contrast, ConfiDx, despite having only 70 billion parameters, successfully identified such uncertainty and outperformed all large-scale models, delivering superior accuracy across the board, ranging from 0.80 to 0.90 (Fig. 4a, b).

### Impact of key factors on performance

**Fig. 5:**
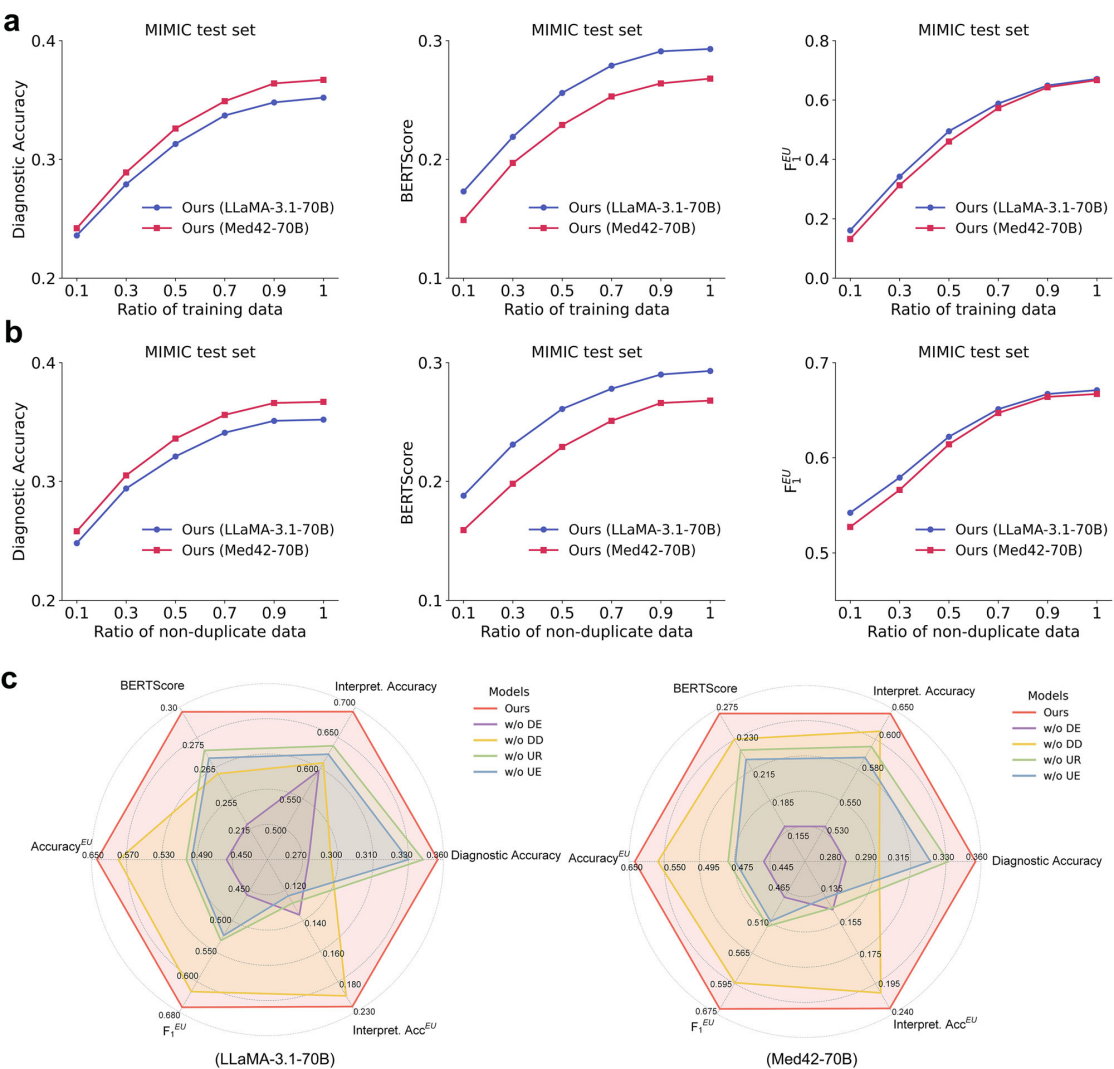
Analysis of key influencing factors. **a** Performance with varying training data size, evaluated by randomly selecting portions of the training data ranging from 10% to 90%. **b** Performance with varying data diversity in training data, achieved by randomly removing portions of the training samples while augmenting the remaining data to maintain a consistent training size. **c** Ablation study performance with different learning objectives in the multi-task learning framework. For this, the learning objective of one task was removed from the current framework, and performance was evaluated across six metrics. DE diagnostic explanation, DD disease diagnosis, UR diagnostic uncertainty recognition, and UE diagnostic uncertainty explanation.

We further investigated the influence of several key factors on the overall performance, including training data size, training data diversity, and the learning objective, on LLaMA-3.1-70B and Med42-70B. To assess the effect of training data size, we randomly selected portions of the training data, ranging from 10% to 90%, and evaluated the performance. As depicted in Fig. 5a, with merely 10% of the training data, the model achieved Diagnostic Accuracy, Interpretation Accuracy, and ${{Accuracy}}^{{EU}}$ scores of 0.239, 0.541, and 0.147, respectively. As the data volume increased, performance improved markedly before gradually converging. To investigate the impact of training data diversity, we randomly removed portions of the training samples while augmenting the remaining data to maintain a consistent training size. As shown in Fig. 5b, when only 10% of the original data was retained, ConfiDx achieved Diagnostic Accuracy, Interpretation Accuracy, and ${{Accuracy}}^{{EU}}$ scores of 0.278, 0.586, and 0.533, respectively. Performance steadily improved with increasing data diversity and similarly converged as the diversity increased. Additional results can be found in Supplementary Note 13. Further, we assessed the impact of the learning objective within the multi-task learning framework. Specifically, we removed one task at a time from the original learning objective and measured the performance across six metrics. The results in Fig. 5c demonstrated that each task within the framework contributes meaningfully to the model’s overall performance.

### Case study

**Fig. 6:**
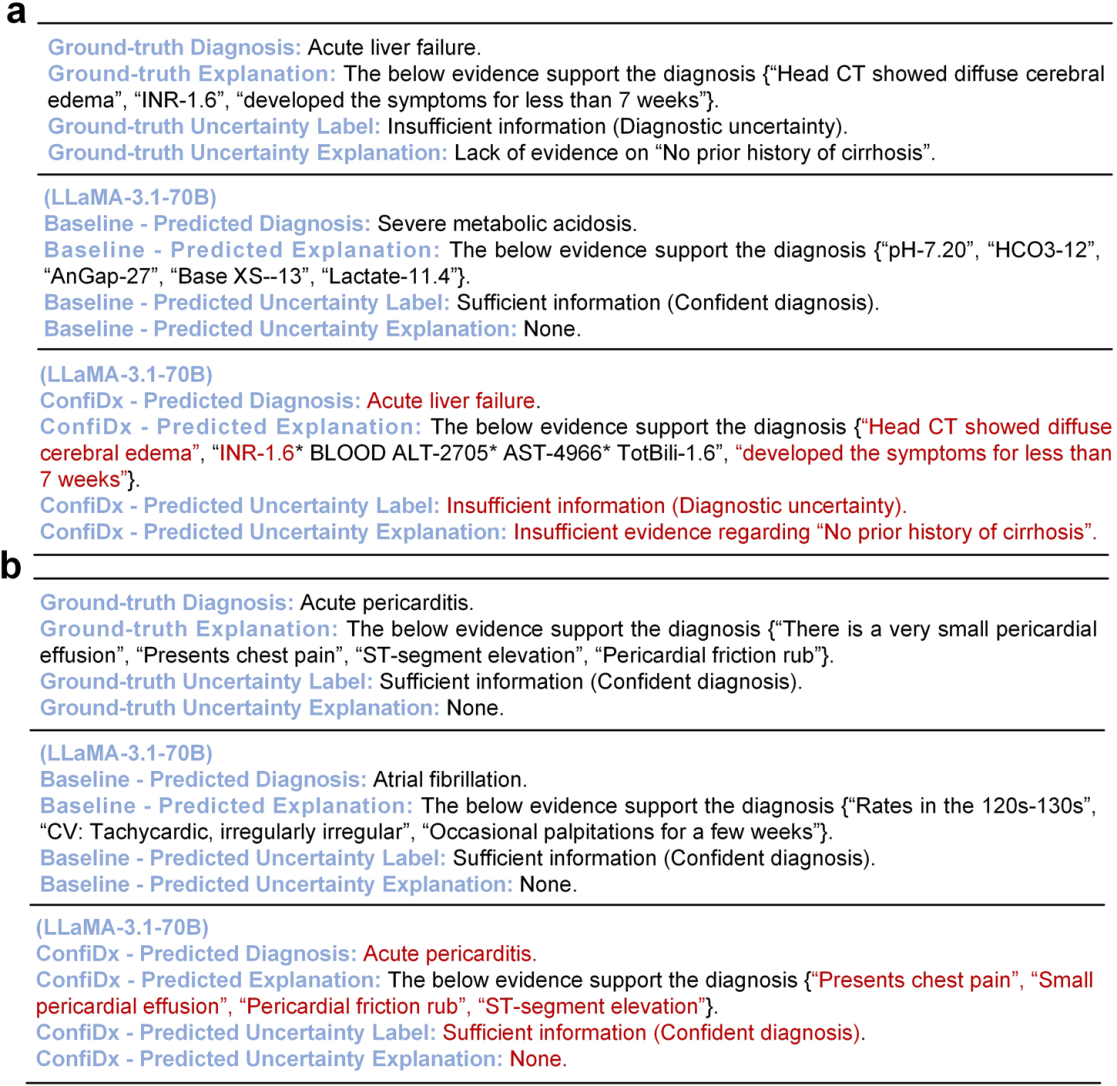
Case studies of off-the-shelf LLaMA-3.1-70B and the fine-tuned counterpart on the MIMIC dataset. Correct predictions are highlighted in red. **a** The note presents incomplete patient information, preventing a definitive diagnosis. **b** The note contains sufficient information for a confident diagnosis.

Case studies were conducted to demonstrate the superior explainability of the fine-tuned LLMs over their off-the-shelf counterpart. As shown in Fig. 6a, off-the-shelf LLaMA produced an incorrect diagnosis and flawed explanations, whereas the fine-tuned model made the correct diagnosis along with three accurate explanations and managed to recognize and explain diagnostic uncertainty. Specifically, since the diagnostic criterion of “no prior history of cirrhosis” was unmet, the processed note primarily indicated acute liver failure but could also, with lower probability, suggest acute decompensation of cirrhosis, thereby leaving the definitive diagnosis uncertain. Notably, the two conditions differ in etiology, severity, treatment, and outcomes; therefore, ConfiDx could eliminate potential risks in clinical practice. Similarly, Fig. 6b illustrates how ConfiDx generated accurate explanations when provided with a clinical note containing sufficient information. Further examples can be found in Supplementary Note 7.

### AI-augmented clinician assessments

**Fig. 7:**
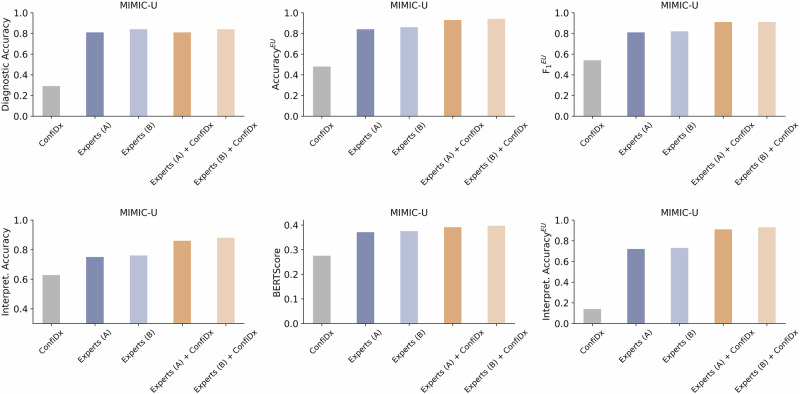
AI-augmented clinician assessments. A performance comparison of ConfiDx, implemented with LLaMA-3.1-70B, medical experts, and model-assisted experts, evaluated on a subset (*n* = 200) of the MIMIC-U dataset. Two groups of clinicians were included: Group A, comprising experts from hepatology and the emergency department, and Group B, comprising cardiology experts. The collaborative approach between AI and medical experts was facilitated by integrating the experts’ diagnostic predictions into the model, which in turn provided its predictions to be considered by the experts during the decision-making process.

We further explored the potential of fine-tuned LLMs in collaboration with medical experts to enhance clinical practice. A total of 200 cases were randomly sampled from the MIMIC-U dataset for manual performance assessment. We implemented the ConfiDx framework using the LLaMA-3.1-70B model and compared the diagnostic performance across three groups: the ConfiDx alone, human experts, and ConfiDx-assisted experts. As shown in Fig. 7, human experts consistently outperformed the standalone LLM across all four diagnostic subtasks. Although ConfiDx did not substantially enhance the diagnostic accuracy of medical experts, AI-augmented experts demonstrated superior performance compared to those working independently on the other subtasks. Specifically, AI-assisted experts (group A) achieved gains of 10.7% in uncertainty recognition, 14.6% in diagnostic explanation accuracy (Interpret. Accuracy), and 26.3% in uncertainty explanation. The overall agreement between the initial diagnoses from experts (group A) and ConfiDx’s predictions was 79%. Specifically, among the 162 correct expert predictions, ConfiDx agreed in 148 cases and disagreed in 14 cases. Among the 38 incorrect expert predictions, ConfiDx agreed in 10 cases and disagreed in 28 cases. These results indicate that ConfiDx used the human input as a reference rather than mirroring it in the human-AI collaboration.

## Discussion

In this study, we developed ConfiDx by fine-tuning open-source LLMs for uncertainty-aware disease diagnosis, addressing the dual challenges of conducting explainable diagnosis and recognizing diagnostic uncertainty due to insufficient evidence in clinical notes. Given the complexity of this task, which involves multiple prediction types, we divided it into four manageable subtasks for performance evaluation. We highlight the following key observations and insights for further discussion.

First, off-the-shelf LLMs demonstrated limited capability in diagnosing diseases, identifying diagnostic uncertainty, and explaining uncertainty across the three datasets (Figs. 2 and 3). The suboptimal performance is unsurprising, considering the challenges posed by real-world clinical notes^2^, which often include extraneous information, such as past medical history, and overlapping disease presentations^3^, such as cardiac arrest and atrial fibrillation. The unsatisfactory performance in identifying diagnostic uncertainty stems from two primary factors: (1) Without pinpointing the most likely diagnosis, LLMs struggle to identify cases with incomplete evidence, as diagnostic criteria differ across diseases. (2) Off-the-shelf LLMs tend to overestimate the sufficiency of clinical note information^17,46^, assuming it contains all necessary evidence for diagnosis, even when this contradicts explicit diagnostic criteria. This overconfidence likely results from their general-purpose training^27,28^, which focuses on simpler tasks like text summarization and question answering. In contrast, real-world disease diagnosis is a complex endeavor requiring customized training to instill the medical expertise needed to follow diagnostic criteria rigorously and seek adequate supporting evidence^2,47^. This discrepancy underscores the necessity of adapting general-purpose LLMs for domain-specific applications to achieve expert-level performance^43^. Furthermore, the inability of off-the-shelf LLMs to explain diagnostic uncertainty is reasonable, as accurate diagnosis and uncertainty recognition are prerequisites for meaningful explanation.

Second, fine-tuned LLMs significantly outperformed their off-the-shelf counterparts in diagnosis and uncertainty recognition on the MIMIC test set (Fig. 2a). The enhanced diagnostic accuracy stems from extensive training data, which allowed the models to learn diverse disease patterns, such as characteristic signs, symptoms, and laboratory results, improving their ability to distinguish between similar conditions. Additionally, the fine-tuning process aligned the models with domain expert behavior by grounding their reasoning in diagnostic criteria. By simulating real-world scenarios where clinical notes lack complete evidence, fine-tuning enabled ConfiDx to assess the sufficiency of the information more effectively, thereby enhancing its ability to recognize uncertainty.

Third, ConfiDx excelled in explanatory capabilities for both diagnosis and uncertainty (Fig. 2b, c) on the MIMIC test set, largely due to the injection of medical knowledge via instruction fine-tuning. By explicitly mapping diagnostic criteria to varied symptom descriptions, the annotated data enabled ConfiDx to understand the meaning of the diagnostic criteria thoroughly. For instance, the models learned to associate diagnostic criteria like “Related symptoms: polyuria, polydipsia, and unexplained weight loss” with variable patient descriptions, such as “three weeks of polyuria and polydipsia” or “10-month history of polyuria with a 15 kg decrease in body weight”. This alignment with diagnostic criteria enabled ConfiDx to identify unmet criteria accurately and clarify the basis for uncertainty.

Additionally, we discovered that ConfiDx exhibited fair robustness in diagnosing diseases, recognizing diagnostic uncertainty, and providing explanations for diseases absent from the training data (Fig. 3a, b). This suggests that the fine-tuning approach effectively leveraged the LLMs’ internal knowledge, grounded in diagnostic criteria, to recognize uncertainty and deliver explanations for unseen cases. Such robustness is particularly significant to clinical applications^16,48^, as it is generally impractical to annotate data for all possible disease types^19^. Encouraging LLMs to effectively leverage the extensive parametric knowledge offers a cost-effective solution in resource-constrained settings.

The fine-tuned LLMs also exhibited superior generalizability, outperforming off-the-shelf models on the external UMN-CDR dataset (Fig. 3c, d). Because the MIMIC training data and the UMN-CDR dataset differ in several critical aspects, such as information recording styles and text length (as outlined in Table 1). This superior generalization underscores the ability of ConfiDx to internalize relevant medical knowledge from diagnostic criteria, enabling its adaptation to clinical notes from diverse healthcare systems.

Several factors influence performance in this study, including the size and diversity of the training data and the choice of learning objectives. Specifically, larger training data exposed the models to a broader spectrum of disease patterns, thus improving diagnostic accuracy^22^ (Fig. 5a). Similarly, more data with detailed evidence-based explanations strengthened the models’ ability to associate diagnostic criteria with varying symptom descriptions, thereby enhancing explanation quality. Besides, data diversity was equally critical (Fig. 5b), as ConfiDx depends on exposure to comprehensive patterns to handle real-world variability^49^. However, performance plateaued with sufficient data coverage (e.g., 90% of the training set, Fig. 5a, b), suggesting diminishing returns from expansions in data size or diversity. Thus, carefully considering data size, diversity, and the cost-effectiveness trade-off is essential when constructing fine-tuning datasets. Additionally, as shown in Fig. 5c, each learning objective contributes to the overall performance in disease diagnosis, uncertainty recognition, and explanation, reflecting the subtasks’ necessity.

We also observed that the fine-tuned LLMs improved medical experts’ ability to recognize diagnostic uncertainty and explain diagnoses (Fig. 7). Although ConfiDx’s diagnostic accuracy was limited by the complexity of real-world clinical notes (Figs. 2a and 3a, c), collaboration between experts and AI addressed this limitation. Experts’ high-accuracy diagnoses were fed into the model, enabling it to retrieve relevant medical knowledge learned from the supervised fine-tuning and improve uncertainty recognition and explanation. This collaboration aids expert decision-making, demonstrating ConfiDx’s potential to assist clinicians in practice.

Despite these advances, our study has the following limitations. First, we did not evaluate smaller LLMs, such as MedAlpaca-7B^50^ or PMCLLaMA-13B^51^, for two key reasons. These models typically have limited in-context lengths, rendering them inadequate for processing real-world clinical notes, which often contain extensive text. Additionally, large-scale models are generally preferred for real-world applications due to their superior performance^1,52^. Smaller models often lack sufficient clinical knowledge or robust instruction-following capabilities^2,53^, limiting their applicability in evidence-based diagnosis. Second, while we observed that closed-source models, such as GPT-4o, also struggled with uncertainty recognition, we were unable to validate the proposed approach on these large-scale commercial models. Third, an alternative human-AI collaboration workflow, in which ConfiDx generates the initial diagnosis for clinician review, may yield complementary insights and warrants future exploration. Lastly, future studies can investigate the effect of fine-tuning in mitigating LLM hallucinations in explainable diagnosis, where hallucinations typically refer to the generation of diagnostic explanations that are factually incorrect.

In summary, our study highlights the critical limitations of current LLMs in recognizing and explaining diagnostic uncertainty. To address this, we formalized the problem of uncertainty-aware disease diagnosis and constructed customized datasets with nuanced annotations for training and evaluation of the trustworthiness of diagnostic models. Besides, we integrated diagnostic criteria into the fine-tuning and developed ConfiDx capable of diagnoses, uncertainty recognition, and delivering explanations. Our fine-tuned LLMs outperformed the off-the-shelf counterparts, achieving superior diagnostic accuracy, more reliable explanations, robust recognition of uncertain cases, and fair generalization in cross-institute evaluations. This study significantly advances the trustworthiness of LLM-based diagnostic models, alleviating concerns about diagnostic uncertainty raised in clinical practice and securing reliable and explainable clinical decision-making.

## Methods

### Definition formulation

Evidence-based diagnosis refers to a diagnosis made by adhering to established diagnostic criteria, utilizing a patient’s clinical information as the basis for decision-making. A confident diagnosis builds on an evidence-based diagnosis, signifying a high degree of certainty in the diagnostic decision. Diagnostic uncertainty arises when diagnostic criteria are unmet due to insufficient evidence, such as the absence of definitive symptoms, ambiguous clinical signs, or inconclusive laboratory findings. Uncertainty-aware disease diagnosis involves developing predictive models that leverage clinical information to determine both the most likely diagnosis and the occurrence of diagnostic uncertainty, distinguishing between confident diagnoses (supported by sufficient evidence) and those characterized by insufficient evidence, while providing detailed explanations.

### Datasets

The MIMIC-IV database^39^ provides de-identified electronic health records (EHRs) for nearly 300,000 patients treated at Beth Israel Deaconess Medical Center in Boston, Massachusetts, USA, from 2008 to 2019. This publicly accessible dataset includes diverse clinical information such as laboratory results, diagnoses, procedures, and unstructured notes like discharge summaries and radiology reports. The UMN-CDR database, a private resource, contains EHRs for ~400,000 patients treated at the University of Minnesota Medical Center, USA, from 2008 to 2022. These records include laboratory results, treatments, and diagnoses, offering extensive clinical information. PubMed Central, maintained by the U.S. National Center for Biotechnology Information, is a free digital archive of biomedical literature. It provides millions of full-text research articles spanning topics like clinical studies, case reports, and systematic reviews, ensuring comprehensive access to scientific publications.

We focused on three clinical specialties linked to critical mortality factors in the USA^42^: endocrinology, cardiology, and hepatology. Diseases were selected through the following process: (1) identifying all diseases within each specialty based on ICD-10-CM codes; (2) excluding diseases without clear diagnostic criteria; (3) removing diseases with fewer than two rules; and (4) excluding diseases not appearing as the primary diagnosis in the selected notes. This yielded 18 disease types, as shown in Supplementary Note 1. The note selection and pre-processing are detailed in Supplementary Note 12. To assess the robustness of LLMs for unseen diseases, we created an independent test set, MIMIC-U, by holding out notes for six diseases with limited notes. The remaining dataset was referred to as MIMIC. For evaluating model generalizability, we extracted notes from the UMN-CDR dataset corresponding to the same disease types as in the MIMIC dataset. This dataset included 11 disease types, excluding acute liver failure. If a disease type has over 100 notes, we randomly sampled 100 notes. From the PMC dataset, we selected 20 real-world case reports tagged as “case report”. Similarly, we manually selected 15 real-world case reports from the NEJM portal. Data statistics of the MIMIC, MIMIC-U, and UMN-CDR are in Table 1.

### Data annotation and split

Diagnostic criteria established by professional associations serve as the gold standard for clinical diagnoses. We manually collected these criteria from authoritative guidelines (detailed in Supplementary Note 8) such as the American Diabetes Association’s criteria for Type 1 Diabetes Mellitus^54^ and the American Heart Association’s criteria for Acute Myocardial Infarction^55^. To simulate real-world scenarios where the ground-truth diagnosis is unavailable, we removed the current diagnosis and, when applicable, excluded medication and discharge details (Supplementary Note 12).

Clinical notes were annotated to evaluate the ability of LLMs to identify diagnostic uncertain cases. Diagnostic criteria served as proxies to determine whether sufficient evidence was present for a confident diagnosis. Specifically, notes containing sufficient evidence aligned with the criteria were labeled as evidence-complete, while those with insufficient evidence were marked as uncertain.

To balance annotation accuracy and efficiency, we employed a multi-agent framework (see Supplementary Note 9) for the majority of the annotation on MIMIC-IV and UMN-CDR datasets, following established methodologies^3,56^. This framework effectively annotated the evidence within the notes, grounded in diagnostic criteria. All relevant descriptions were annotated as diagnostic evidence to produce evidence-complete notes. Evidence-incomplete notes were generated by masking portions of the evidence, simulating incomplete clinical information. Domain experts reviewed the modified notes to ensure that masking did not alter the diagnosis. We also sampled a subset of notes for human annotation to evaluate inter-annotator agreement (IAA) between the LLM and human annotations (Supplementary Note 9). For the PMC dataset, two experts reviewed each report, extracted diagnoses, and annotated all evidence supporting the diagnosis based on diagnostic criteria.

Figure 1 illustrates the data split, with notes maintaining a 1:1 ratio of evidence-complete to incomplete cases. The dataset was randomly divided into training, validation, and test sets in a 7:1:2 proportion while preserving this balance across all subsets.

### Comparison with other large-scale LLMs

We aim to verify whether advanced commercial LLMs with hundreds of billions of parameters perform well in uncertainty-aware diagnosis and compare them with our fine-tuned 70B-parameter LLMs. Due to privacy concerns, we assessed model performance on publicly available case reports from the PMC portal. We tested five state-of-the-art LLMs, GPT-4o (gpt-4o-2024-08-06), OpenAI-o1 (o1-2024-12-17), Gemini-2.0 (gemini-2.0-flash), and Claude-3.7 (claude-3-7-sonnet-20250219), and DeepSeek-R1, focusing on diagnostic uncertainty recognition. For a fair comparison, each model was provided with the ground-truth diagnosis and corresponding diagnostic criteria, mirroring the data annotation process. Using zero-shot prompting (Supplementary Note 10), we instructed the LLMs to determine whether the clinical notes contained sufficient evidence for a confident diagnosis. A clinician manually reviewed all predictions.

### ConfiDx

We developed ConfiDx on open-source LLMs for explainable diagnosis via instruction fine-tuning, where diagnostic tasks were formatted as natural language instructions with structured outputs. Clinical notes were converted into instructional demonstrations, each comprising: (1) an input note with patient information, (2) a task instruction, and (3) an output containing the diagnosis, explanation, and uncertainty label.

A key challenge arose from the need to process complex, multi-component instructions for simultaneous prediction of diagnosis, explanation, and uncertainty. To address this, we decomposed the task into four subtasks: (1) disease diagnosis, (2) diagnostic explanation, (3) uncertainty recognition, and (4) uncertainty explanation. This decomposition enabled more effective processing through a multi-task learning framework, with separate instruction sets for each component (Supplementary Note 11). For optimization, we used cross-entropy loss for diagnosis and uncertainty recognition. For the explanation generation, we employed soft F1 loss^57,58^ to account for potential discrepancies between the number of explanatory components in predictions and references, and the need for an item-by-item comparison.

We adopted four widely used open-source LLMs for training and evaluation on real-world clinical notes: two general-domain models, LLaMA-3.1-70B-Instruct (LLaMA-3.1-70B) and DeepSeek-R1-Distill-Llama-70B^45^ (DeepSeek-R1-70B), and two domain-specific models, BioLLaMA-70B and Med42-70B^59^.

We selected models with 70 billion parameters to balance computational efficiency and predictive performance, as clinicians often favor larger models for their reliability and trustworthy predictions in complex diagnostic scenarios^1,52,60^. We implemented parameter-efficient fine-tuning via Low-Rank Adaptation (LoRA)^61^ using the Hugging Face framework. Experiments were conducted with a batch size of 4, 10 epochs, a maximum learning rate of 1e−4, and a 0.01 warm-up ratio. LoRA parameters included a rank of 8, alpha of 16, and a dropout rate of 0.05. No further hyperparameter tuning was performed. Fine-tuning and inference were performed on 10 Nvidia A100 GPUs (80 GB VRAM) with input data and instructions provided during inference.

### Key factors analysis

To evaluate the impact of training data size, we varied the training set from 10% to 90% of the data for fine-tuning, with notes randomly selected while stratifying by disease type. For data diversity analysis, we reduced diversity by retaining only 10–90% of the data, stratified by disease type, and duplicating the remaining notes to restore the original dataset size. This process ensured the data size remained consistent despite reduced diversity. To analyze the effect of learning objectives, we excluded one task at a time from the original objective, fine-tuned the model with the modified objective, and evaluated performance.

### Integration of ConfiDx and clinicians

To explore the potential of human-machine collaboration in enhancing clinical decision-making, we integrated the fine-tuned LLMs with medical experts. In this collaborative framework, the experts’ diagnostic predictions were input into the model, and the model’s outputs were subsequently presented to the experts for further evaluation. This design enabled us to highlight ConfiDx’s strengths in diagnostic interpretation and in recognizing and explaining uncertainty. Specifically, to avoid simply mirroring the experts’ initial diagnosis, we carefully designed the prompts (see Supplementary Note 2). Two groups of clinicians were involved: Group A, consisting of two experts from hepatology and the emergency department, and Group B, comprising clinicians from cardiology. The clinicians, each with internet access, participated in the process, generating consensus-based predictions. For performance assessment, we randomly selected 200 samples from the MIMIC-U dataset, stratified by disease type.

### Automatic evaluation metrics

Following related works^1,2,62,63^, we evaluated diagnostic performance with accuracy and the ability to correctly identify uncertainty with ${{Accuracy}}^{{EU}}$ and ${F}_{1}^{{EU}}$ score. The computation of ${{Accuracy}}^{{EU}}$ is as follows:

1 $${{Accuracy}}^{{EU}}=\frac{{\rm{Cumulative\; number\; of\; correctly\; recognized\; evidence\; uncertainty}}}{{\rm{Total\; number\; of\; evidence\; uncertainty}}}$$

The computation of ${F}_{1}^{{EU}}$ score is shown below:

2 $${\rm{Precision}}=\frac{{\rm{TP}}}{{\rm{TP}}+{\rm{FP}}}$$

3 $${\rm{Recall}}=\frac{{\rm{TP}}}{{\rm{TP}}+{\rm{FN}}}$$

4 $${F}_{1}^{{EU}}=\frac{2\times {\rm{Precision}}\times {\rm{Recall}}}{{\rm{Precision}}+{\rm{Recall}}}$$

where TP (true positives) denotes uncertainty cases correctly identified as uncertain, FP (false positives) denotes cases incorrectly identified as uncertain, and FN (false negatives) refers to missed or unrecognized uncertainty cases. Regarding explanation evaluation, we adopted METEOR^64^, BERTScore^65^, SentenceBert^66^, and Interpretation Accuracy^3^ since these metrics have been widely used to measure the semantic similarity between the reference and generated text^67,68^. Concretely, METEOR^64^ primarily focused on surface-level similarities by leveraging n-gram overlap and linguistic features like stemming and synonyms, while BERTScore^65^ and SentenceBert^66^ assessed semantic similarity using contextual embeddings from pre-trained language models. The Interpretation Accuracy^3^ is computed as:

5 $${\rm{Interpret}}.{\rm{Accuracy}}=\frac{{\rm{Cumulative\; number\; of\; correct\; explanations}}}{{\rm{Total\; number\; of\; explanations}}}$$

Similarly, we measured the alignment between the generated explanations and ground-truth with ${Interpret}.\,{{Acc}{uracy}}^{{EU}}$ using the formula:

6 $${Interpret}.\,{{Accuracy}}^{{EU}}=\frac{{\rm{Cumulative\; number\; of\; correct\; explanations\; for\; incomplete\; evidence}}}{{\rm{Total\; number\; of\; explanations\; for\; incomplete\; evidence}}}$$

### Manual evaluation

We conducted a manual evaluation of the predicted explanations. For diagnostic explanations, we randomly selected 100 notes from the MIMIC test set, evenly split between evidence-complete and evidence-incomplete notes. For uncertainty recognition explanations, we randomly sampled 100 evidence-incomplete notes from the same source. Following prior work^43,44^, we assessed explanation quality along two dimensions: correctness, reflecting medical accuracy, and completeness, measuring how comprehensively symptom descriptions were addressed. Each metric was rated on a 5-point scale. A score of 5 indicated that over 80% of ground-truth explanations were accurately predicted (correctness) or that more than 80% of explanations matched the ground-truth (completeness). A score of 1 denoted less than 20% accuracy or completeness. Two physicians independently graded each note, with a third resolving any discrepancies.

### Statistical analysis

We used the non-parametric bootstrap procedure with 200 iterations to estimate the mean values and 95% confidence intervals for the evaluation metrics. For each bootstrap iteration, a resampled dataset of the same size as the test set was generated through random sampling with replacement.

## Supplementary information

Supplementary Data_R1_0912

Supplementary Note 8

## Data Availability

The MIMIC-IV dataset can be found at https://physionet.org/content/mimiciv/3.1/ and requires access due to its terms of use. UMN-CDR was sourced from real-world clinical scenarios, with IRB approval. Due to privacy regulations, the clinical notes cannot be released to the public. PMC case reports can be found at https://www.ncbi.nlm.nih.gov/pmc and https://github.com/pmc-patients/pmc-patients. NEJM case reports can be found at https://www.nejm.org/browse/nejm-article-category/clinical-cases. The compiled diagnostic criteria, manually annotated PMC case reports, and NEJM case reports are released at https://github.com/betterzhou/ConfiDx.

## Code availability

The codes are available for scientific research and non-commercial use at https://github.com/betterzhou/ConfiDx.
