# Supplementary material for: Uncertainty-aware large language models for explainable disease diagnosis
Source: NPJ Digit Med. 2025 Nov 18;8:690. doi: 10.1038/s41746-025-02071-6 (PMC12627459; doi:10.1038/s41746-025-02071-6)
Supplement: Supplementary file 1 — Supplementary Data_R1_0912 [file 41746_2025_2071_MOESM1_ESM.pdf]

Supplementary Note 1

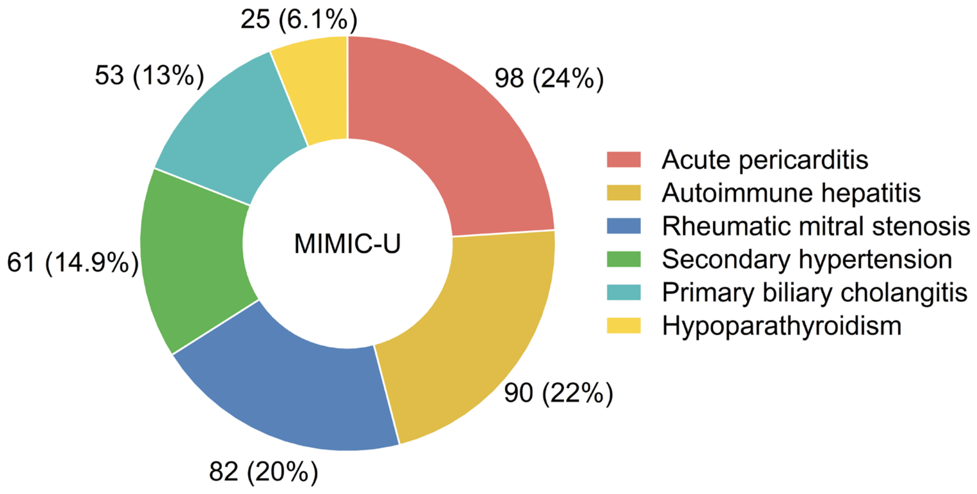

Supplementary Figure 1. Data composition of the constructed MIMIC-U dataset.

Supplementary Table 1. Overview of the included diseases in the MIMIC-IV dataset.

Index	Disease	ICD-10-CM Code	Note Number
1	Hyperthyroidism	E05	470
2	Type 1 diabetes mellitus	E10	927
3	Type 2 diabetes mellitus	E11	4,294
4	Hypoparathyroidism	E20	25
5	Hyperfunction of pituitary gland	E22	685
6	Rheumatic mitral stenosis	I05.0	82
7	Rheumatic tricuspid insufficiency	I07.1	264
8	Essential (primary) hypertension	I10	6,996
9	Secondary hypertension	I15	61
10	Hypertensive crisis	I16	409
11	Acute myocardial infarction	I21	656
12	Chronic ischemic heart disease	I25	3,085
13	Acute pericarditis	I30	98
14	Cardiac arrest	I46	308
15	Heart failure	I50	5,794
16	Acute liver failure	K72.0	479
17	Primary biliary cholangitis	K74.3	53
18	Autoimmune hepatitis	K75.4	90

Supplementary Table 2. Overview of the included diseases in the UMN-CDR dataset.

Index	Disease	ICD-10-CM Code	Note Number
1	Hyperthyroidism	E05	100
2	Type 1 diabetes mellitus	E10	100

3	Type 2 diabetes mellitus	E11	100
4	Hyperfunction of pituitary gland	E22	64
5	Rheumatic tricuspid insufficiency	I07.1	47
6	Essential (primary) hypertension	I10	100
7	Hypertensive crisis	I16	59
8	Acute myocardial infarction	I21	100
9	Chronic ischemic heart disease	I25	100
10	Cardiac arrest	I46	38
11	Heart failure	I50	100

Supplementary Note 2

In this section, we introduced the prompts used for LLM inference in disease diagnosis and diagnostic uncertainty recognition. Since uncertainty recognition depends on diagnostic criteria and off-the-shelf LLMs may lack or only know partial knowledge, we provided the diagnostic criteria for the baseline models to ensure a fair comparison. Notably, during inference, diagnostic criteria were not supplied to the fine-tuned LLMs.

To emulate real-world diagnostic scenarios where ground-truth diagnoses remain unknown, we refrained from directly providing the diagnostic criteria of the ground-truth diagnoses. Instead, we supplied the diagnostic criteria of the predicted diagnoses. However, since predicted diagnoses may span arbitrary diseases and our collected criteria only cover the selected diseases, the criteria were provided to off-the-shelf LLMs only when the predicted diagnosis matched one of the selected diseases.

In the human-AI collaboration experiments, the prompts for disease diagnosis prediction differed from the standard setup, while the prompts for all other tasks remained unchanged.

Prompts for disease diagnosis prediction:

You are an experienced doctor. Given a patient's clinical note {...}, please use step-by-step deduction to identify the most likely disease. Notably, only provide the predicted disease without including your rationale. The output should be a Python list object with the format of [""].

Prompts for disease diagnosis prediction (Human-AI collaboration):

You are an experienced doctor. Given a patient's clinical note {...} and a predicted diagnosis {...}, please use step-by-step deduction to identify the most likely disease. NOTABLY, THE PROVIDED DIAGNOSIS MIGHT NOT NECESSARILY BE CORRECT. YOU SHOULD MAKE AN INDEPENDENT PREDICTION. Only provide the predicted disease without including your rationale. The output should be a Python list object with the format of [""].

Prompts for diagnostic uncertainty recognition:

You are an experienced doctor. Given a patient's clinical note {...}, the predicted diagnosis {...}, and the corresponding diagnostic criteria {...}, please use step-by-step deduction to determine whether the clinical note provides sufficient information to satisfy all the entries in the diagnostic criteria. Ensure that your assessment strictly adheres to the diagnostic criteria. The output should be a Python list object in the format: [""]. If the clinical note meets all the criteria, output ["sufficient information"]. Otherwise, output ["incomplete information"].

Supplementary Note 3

In this section, we introduce the prompts used for LLM inference on diagnosis explanation and diagnostic uncertainty explanation. Considering that the two tasks depend on diagnostic criteria and off-the-shelf LLMs may lack or only know partial knowledge, we provided the diagnostic criteria for the baseline models to ensure a fair comparison. Notably, during inference, diagnostic criteria were not supplied to the fine-tuned LLMs.

To simulate the real-world diagnosis scenarios where ground-truth diagnoses are unknown, we refrained from directly providing the diagnostic criteria of the ground-truth diagnoses. Instead, we supplied the diagnostic criteria of the predicted diagnoses. However, since predicted diagnoses may span arbitrary diseases, and our collected criteria only cover the selected diseases, the criteria were provided to off-the-shelf LLMs only when the predicted diagnosis matched one of the selected diseases.

Prompts for diagnostic explanation:

You are an experienced doctor. Given a patient's clinical note {...} and the predicted diagnosis {...}, and the corresponding diagnostic criteria {...}, please explain which pieces of patient information from the note support the diagnosis. Please note that your explanation should be grounded in the diagnostic criteria and follow a step-by-step deductive reasoning process. Use only the information provided in the clinical note as diagnostic evidence without altering its wording. The output should be a Python list object with the format of ["", "", ""]. Notably, each piece of evidence is a separate item enclosed in double quotation marks.

Prompts for diagnostic uncertainty explanation:

You are an experienced doctor. You will be provided with a patient's clinical note {...}, the predicted diagnosis {...}, and the identified evidence {...}. However, the current evidence is incomplete to fully support the diagnosis. Please strictly follow the disease's diagnostic criteria {...} to clarify which criteria remain unmet. Ensure that your explanation is grounded in the diagnostic criteria and follows a step-by-step deductive reasoning process. Only list the unmet criteria without providing additional rationales. The output should be a Python list object with the format of [""].

Supplementary Note 4

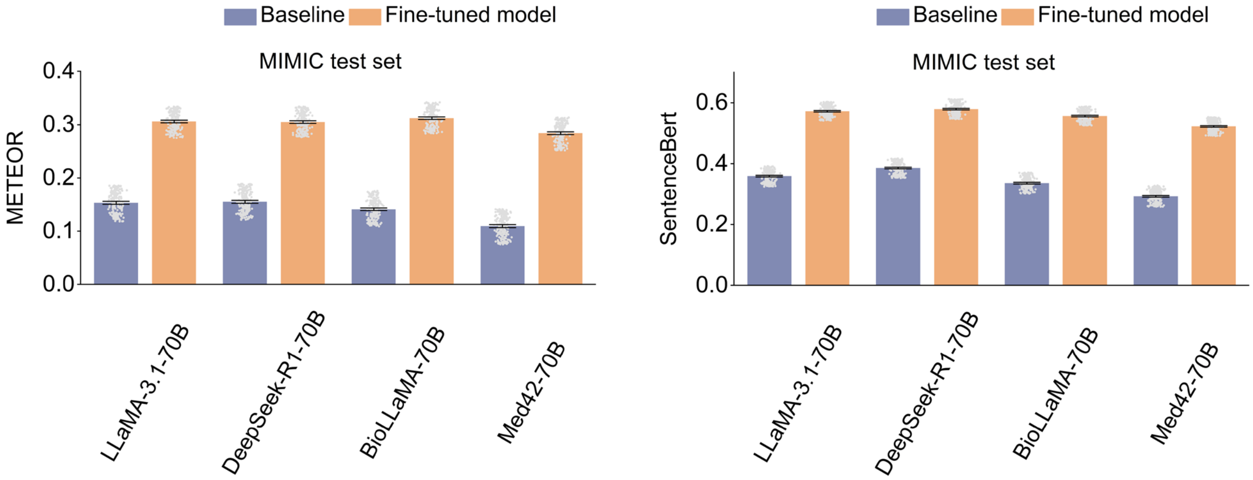

Supplementary Figure 2. Automatic evaluation of the explanations for disease diagnosis on the test set of the MIMIC dataset (using metrics METEOR and SentenceBert). Error bars represent the 95% CI of the mean, calculated via bootstrapping.

Supplementary Note 5

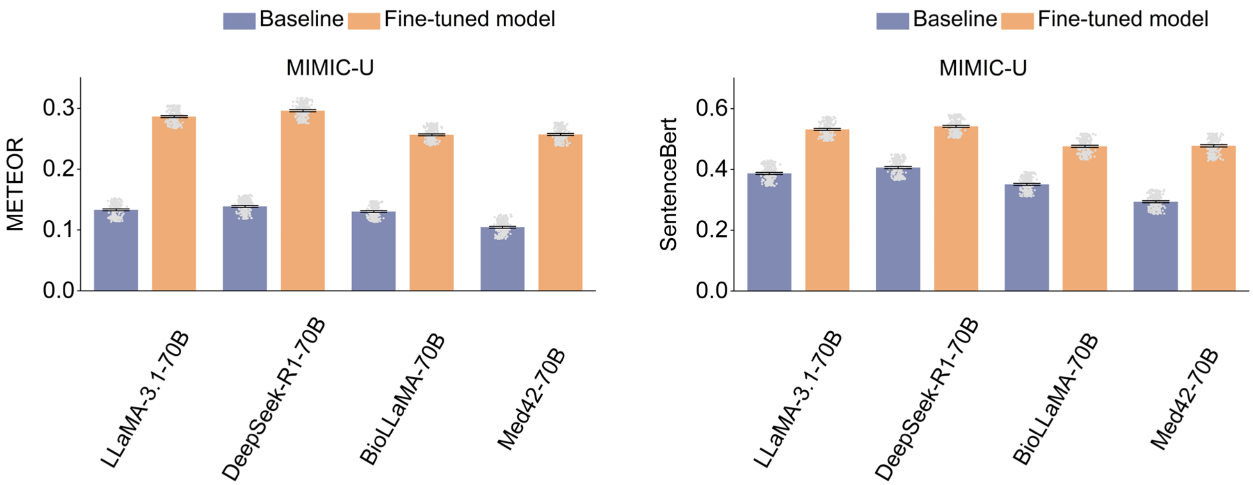

Supplementary Figure 3. Automatic evaluation of the explanations for disease diagnosis on the MIMIC-U dataset with hold-out disease types (using metrics METEOR and SentenceBert). Error bars represent the 95% CI of the mean, calculated via bootstrapping.

Supplementary Note 6

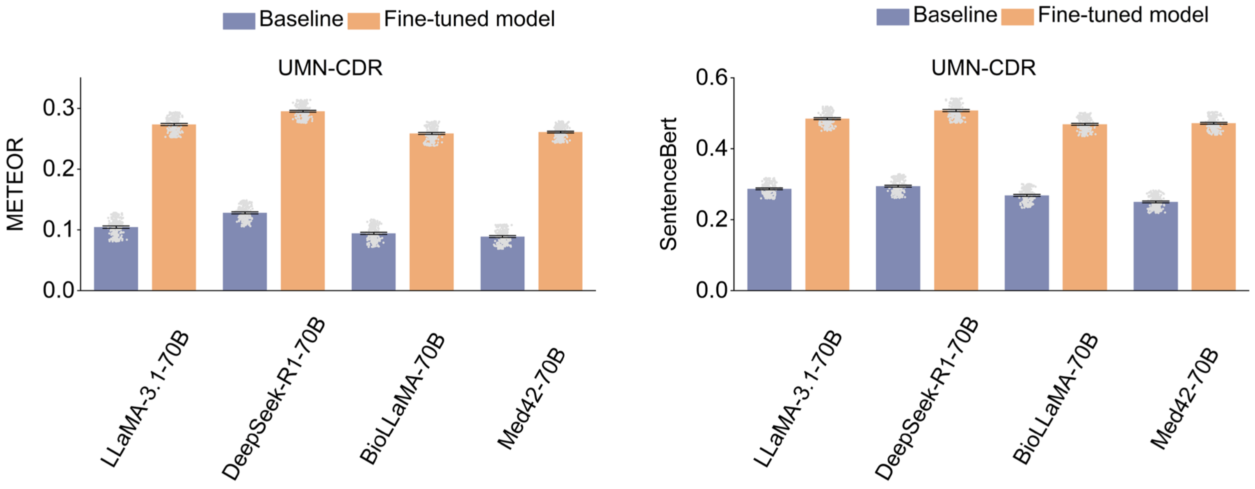

Supplementary Figure 4. Automatic evaluation of the explanations for disease diagnosis on the external UMN-CDR dataset (using metrics METEOR and SentenceBert). Error bars represent the 95% CI of the mean, calculated via bootstrapping.

Supplementary Note 7

Ground-truth Diagnosis: Acute liver failure.
Ground-truth Explanation: The below evidence support the diagnosis {"Acute Encephalopathy: Anisocoria (L)", "Serum (blood) INR >=1.5", "Current illness < 26 weeks duration"}.
Ground-truth Uncertainty Label: Insufficient information (Diagnostic uncertainty).
Ground-truth Uncertainty Explanation: Lack of evidence on "No prior history of cirrhosis".

(LLaMA-3.1-70B)
Baseline - Predicted Diagnosis: Non-ischemic cardiomyopathy.
Baseline - Predicted Explanation: The below evidence support the diagnosis {"Prior history of non-ischemic cardiomyopathy", "progressive DOE", "TTE with LVEF 20%, RV dysfunction", "Increase in MRTR"}.
Baseline - Predicted Uncertainty Label: Sufficient information (Confident diagnosis).
Baseline - Predicted Uncertainty Explanation: None.

(LLaMA-3.1-70B)
ConfiDx - Predicted Diagnosis: Acute liver failure.
ConfiDx - Predicted Explanation: The below evidence support the diagnosis {"Acute Encephalopathy: Anisocoria", "Thrombocytopenia and hemorrhage", "Serum (blood) INR >=1.5", "ALT(SGPT)-462, AST(SGOT)-626, LD(LDH)-1122"}.
ConfiDx - Predicted Uncertainty Label: Sufficient information (Confident diagnosis).
ConfiDx - Predicted Uncertainty Explanation: None.

Supplementary Figure 5. Case study of off-the-shelf LLaMA-3.1-70B and the fine-tuned counterpart on the MIMIC test set. Correct predictions are highlighted in blue.

Ground-truth Diagnosis: Type 1 diabetes mellitus.
Ground-truth Explanation: The below evidence support the diagnosis {"polyuria, polydipsia", "a random plasma glucose 210 mg/dL"}.
Ground-truth Uncertainty Label: Insufficient information (Diagnostic uncertainty).
Ground-truth Uncertainty Explanation: Lack of evidence on "Laboratory test: Presence of islet autoantibodies, such as glutamic acid decarboxylase antibodies (GAD), islet cell antibodies (ICA)".

(Med42-70B)
Baseline - Predicted Diagnosis: Preeclampsia.
Baseline - Predicted Explanation: The below evidence support the diagnosis {"urine P:C of 0.3", "PROTEIN-30", "URIC ACID-4.3"}.
Baseline - Predicted Uncertainty Label: Sufficient information (Confident diagnosis).
Baseline - Predicted Uncertainty Explanation: None.

(Med42-70B)
ConfiDx - Predicted Diagnosis: Type 1 diabetes mellitus.
ConfiDx - Predicted Explanation: The below evidence support the diagnosis {"polyuria, polydipsia", "GLUCOSE-70", "a random plasma glucose 210 mg/dL", "she was initially given an insulin drip intrapartum and was transitioned to her home insulin pump postpartum"}.
ConfiDx - Predicted Uncertainty Label: Insufficient information (Diagnostic uncertainty).
ConfiDx - Predicted Uncertainty Explanation: Insufficient evidence regarding "Laboratory test: Presence of islet autoantibodies".

Supplementary Figure 6. Case study of off-the-shelf Med42-70B and the fine-tuned counterpart on the MIMIC test set. Correct predictions are highlighted in blue.

Supplementary Note 8

We provided a comprehensive overview of the diagnostic criteria, citing their respective sources, in the attached Excel file.

For diseases with subgroup-specific criteria, such as those tailored to pregnancy or pediatric cases, we focused exclusively on criteria applicable to the general population. As a result, clinical notes of these subgroups (e.g., pregnancy or pediatric cases) were excluded from the final dataset (see Supplementary Note 12).

Supplementary Note 9

In this section, we detail the multi-agent framework designed for diagnostic explanation annotation and introduce the assessment of annotation quality with human effort.

The core idea of explanation annotation is to associate diagnostic criteria with corresponding descriptions in clinical notes, identifying relevant evidence as diagnostic explanations. The framework consists of four LLM-based evidence extractors, one LLM-based examiner, and one LLM-based generator. The evidence extractors identify relevant evidence, the examiner consolidates and verifies the extracted evidence, and the generator supplements missing information in the clinical notes.

Specifically, each evidence extractor is prompted to iteratively review each diagnostic criterion and locate the related descriptions in the clinical notes. For instance, a diagnostic rule such as "Related symptoms: polyuria, polydipsia, unexplained weight loss" might correspond to a description like "a 10-month history of polyuria" in the note. In more complex cases, where an entry matches patient information dispersed across multiple sentences, we annotated all related descriptions as the ground-truth evidence for that entry. For example, if the criterion matched two separate descriptions, such as "a 10-month history of polyuria" and "weight loss of 15 kilograms", we concatenated these sentences to form the final evidence for that entry.

Once the evidence extraction is completed, all outputs are passed to the LLM-based examiner for verification. The examiner performs two key tasks: first, it integrates the extracted evidence from the four extractors to determine the final annotation; second, it assesses whether the clinical note includes all required components (e.g., symptoms, signs, or laboratory test results) specified by the diagnostic criteria. If any essential information is missing, the examiner instructs the generator to produce the corresponding information to supplement the note.

Regarding implementation, two of the evidence extractors, along with the examiner and generator, are built using LLaMA-3.1-70B, while the remaining two extractors utilize DeepSeek-R1-70B. To ensure consistency and variety, the temperature parameter for the LLM-based extractors is set to 0.1, while it is set to 1 for the LLM-based generator. The prompts used for the extractors, examiner, and generator are provided below.

Prompts for the LLM-based evidence extractors:

You are an experienced doctor. Given the clinical note {...}, the diagnosis {...}, and an entry {...} of the diagnostic criteria, please find the most related description from the note as diagnostic evidence that corresponds to this entry. Please note that you should distinguish the patient's recent and historical information. Notably, the note may contain multiple descriptions related to this entry but are written in different sentences. In such cases, you should output all the closely related descriptions. Additionally, the note may not contain any related evidence that corresponds to this entry. In such cases, you should only find the closely related description corresponding to the input entry. Finally, if you can find such evidence, the output is the identified evidence. If not, the output is ["None"]. The output should be a Python list object with the format of [""] or ["", ""].

Prompts for the LLM-based examiner:

You are an experienced doctor. Given the clinical note {...}, the diagnosis {...}, an entry {...} of the diagnostic criterion, and the identified diagnostic evidence from the note {...}, please determine which identified evidence could best be aligned with the diagnostic entry. Please note that you should distinguish the patient's recent and historical information. Notably, the note may

contain multiple descriptions related to this entry but are written in different sentences. In such cases, you should output all the closely related descriptions. Additionally, the note may not contain any related evidence that corresponds to this entry. In such cases, you should only find the closely related description corresponding to the input entry. Finally, if you can find such evidence, the output is the identified evidence. If you find multiple pieces of evidence, please concatenate the evidence into a single sentence. If the note does not contain any related evidence, the output is ["None"]. The final output should be a Python list object with the format of [""].

Prompts for the LLM-based generator:
You are an experienced doctor and need to generate a symptom description to supplement a patient's clinical note. Given the clinical note {...}, the diagnosis {...}, and an entry {...} of the diagnostic criterion, please generate a piece of symptom description that can closely align to the diagnostic entry. You should also consider the patient's status and profiles. The final output should be a Python list object with the format of [""].

Annotation quality assessment with human effort:
In this study, we assessed the quality of annotations for diagnostic uncertainty and explanation by calculating the inter-annotator agreement (IAA) score between an LLM annotator and two human annotators. Cohen's Kappa statistic (κ) was utilized to quantify annotation agreement, as it accounts for the likelihood of agreement occurring by chance. The Kappa statistic ranges from 0 (chance-level agreement) to 1 (perfect agreement). The annotation process involved three domain experts: Annotator 1 (A1), a registered nurse; Annotator 2 (A2), a medical student; and Annotator 3 (A3), a graduate student in nursing school. To ensure consistency, all human annotators received standardized instructions and example annotations before commencing their work. For each clinical note, individual diagnostic criteria were iteratively reviewed. Agreement between the LLM and human annotations was scored as 1 if both aligned and 0 if they diverged. For diagnostic explanations, which often involve multiple entries, agreement was assessed for each entry, and the cumulative agreement score was calculated. Annotator 3 acted as an adjudicator to assess the consistency between the human and LLM annotations.
We calculated Cohen's Kappa statistic separately for annotations of diagnostic uncertainty and explanation. For this analysis, 100 clinical notes were sampled from the MIMIC and UMN datasets for each task. Each annotation was alternately treated as the ground truth while the other served as the prediction, and the final κ score was computed as the average across these iterations. The results demonstrated a high level of agreement between the LLM and human annotators. Specifically, for diagnostic uncertainty, the average Kappa scores were 0.93 for MIMIC and 0.91 for UMN. Regarding diagnostic explanation, the average Kappa scores were 0.714 for MIMIC and 0.695 for UMN. These results indicate strong consistency and reliability of the proposed multi-agent framework in annotating diagnostic uncertainty and reasonable agreement in annotating diagnostic explanations.

Supplementary Note 10

In this section, we introduce the details of prompting large-scale commercial LLMs on the PMC case reports and NEJM case reports. The purpose is to verify whether advanced commercial LLMs with hundreds of billions of parameters perform well in uncertainty-aware diagnosis. Since uncertainty recognition depends on diagnostic criteria and off-the-shelf LLMs may lack or only know partial knowledge, we provided the diagnostic criteria of ground-truth diagnosis for the off-the-shelf LLMs to ensure a fair comparison. Notably, during inference, diagnostic criteria were not supplied to the fine-tuned LLMs.

Prompts for uncertainty recognition on the PMC/NEJM case reports:
The correct diagnosis of the case is {...}. The diagnostic criteria for this disease are as follows: {...}. Please use step-by-step deduction to determine whether the clinical note provides sufficient information to satisfy all the entries in the diagnostic criteria. Ensure that your assessment strictly adheres to the diagnostic criteria. The output should be a Python list object in the format: [""]. If the clinical note meets all the criteria, output ["sufficient information"]. Otherwise, output ["incomplete information"].

Supplementary Note 11

In this study, we proposed splitting the long instructions into four smaller, more manageable parts. Each part focused on a specific task: disease diagnosis, diagnostic explanation, recognition of diagnostic uncertainty, and explanation of diagnostic uncertainty. To facilitate this, we designed a multi-task learning framework that utilized the four distinct sets of annotated data for instruction fine-tuning. An example of instructional demonstrations for each task is presented below. Generally, each training instance has three components: (1) an instruction describing the task to perform, (2) an input data that describes the patient's information or other related information, e.g., clinical note, and (3) an output that contains the ground-truth, such as diagnosis, diagnostic explanations, or uncertainty label.

Instructional demonstration example for disease diagnosis:

**Instruction:**<br>*You are an experienced doctor. Given a patient's clinical note, please use step-by-step deduction to identify the most likely disease. Notably, only provide the predicted disease without including your rationale. The output should be a Python list object with the format of [""].*
**Input:**<br>*Here is the patient's clinical note: {"A 49-year-old woman …"}*
**Output:**<br>*["Acute pericarditis"]*

Instructional demonstration example for diagnostic explanation:

**Instruction:**<br>*You are an experienced doctor. Given a patient's clinical note and the predicted diagnosis, and the corresponding diagnostic criteria, please explain which pieces of patient information from the note support the diagnosis. Please note that your explanation should be grounded in the diagnostic criteria and follow a step-by-step deductive reasoning process. Use only the information provided in the clinical note as diagnostic evidence without altering its wording. The output should be a Python list object with the format of ["", "", ""]. Notably, each piece of evidence is a separate item enclosed in double quotation marks.*
**Input:**<br>*Here is the patient's clinical note: {"A 49-year-old woman …"}*<br><br>*Here is the predicted diagnosis: {"Acute pericarditis"}*<br><br>*Here are the diagnostic criteria: {"Typical chest pain", …}*
**Output:**<br>*["There is a very small pericardial effusion", "presents chest pain", "ST-segment elevation"]*

Instructional demonstration example for diagnostic uncertainty recognition:

**Instruction:**<br>*You are an experienced doctor. Given a patient's clinical note, the predicted diagnosis, and the corresponding diagnostic criteria, please use step-by-step deduction to determine whether the*

clinical note provides sufficient information to satisfy all the entries in the diagnostic criteria. Ensure that your assessment strictly adheres to the diagnostic criteria. The output should be a Python list object in the format: [""]. If the clinical note meets all the criteria, output ["sufficient information"]. Otherwise, output ["incomplete information"].
**Input:**<br>*Here is the patient's clinical note: {"A 49-year-old woman …"}*<br><br>*Here is the predicted diagnosis: {"Acute pericarditis"}*<br><br>*Here are the diagnostic criteria: {"Typical chest pain", …}*
**Output:**<br>*["incomplete information"]*

Instructional demonstration example for diagnostic uncertainty explanation:

**Instruction:**<br>*You are an experienced doctor. You will be provided with a patient's clinical note, the predicted diagnosis, and the identified evidence. However, the current evidence is incomplete to fully support the diagnosis. Please strictly follow the disease's diagnostic criteria to clarify which criteria remain unmet. Ensure that your explanation is grounded in the diagnostic criteria and follows a step-by-step deductive reasoning process. Only list the unmet criteria without providing additional rationales. The output should be a Python list object with the format of [""].*
**Input:**<br>*Here is the patient's clinical note: {"A 49-year-old woman …"}*<br><br>*Here is the predicted diagnosis: {"Acute pericarditis"}*<br><br>*Here is the identified evidence: ["There is a very small pericardial effusion", …]*<br><br>*Here are the diagnostic criteria: {"Typical chest pain", …}*
**Output:**<br>*[Insufficient evidence regarding "Pericardial friction rub"].*

Supplementary Note 12

In this section, we outline the processes of note selection and preprocessing for the MIMIC-IV and UMN-CDR datasets.

Our analysis focuses on diseases from three clinical specialties: endocrinology, cardiology, and hepatology. To ensure that the selected diseases represented the primary diagnoses for the patient's hospital visit, we employed a rigorous filtering strategy. Relevant patients were initially identified by filtering the diagnosis table, which contains all recorded ICD codes. This was followed by a detailed review of each patient's discharge summary, retaining only those cases where the initial primary diagnosis corresponded to one of the target pathologies. Patients with diagnoses spanning more than one of the selected diseases were excluded to avoid confounding factors. Given that certain diseases involve subgroup-specific diagnostic criteria, such as those applicable to pregnancy or pediatric cases, we focused solely on criteria developed for the general population. Consequently, we retained only the clinical notes of patients to whom the selected diagnostic criteria were applicable. To accommodate the input context limitations of the adopted open-source LLMs, we also excluded clinical notes exceeding a specified length. A summary of the data statistics for the selected notes is presented in Table 1 of the manuscript.

Clinical note preprocessing was conducted to simulate real-world scenarios where the ground-truth diagnosis is unavailable while preserving relevant historical patient information. To achieve this, we removed the current diagnosis and, when applicable, excluded medication and discharge details. Four complementary strategies were applied. First, section-level removal: open-source LLMs (LLaMA-3.1-70B and DeepSeek-R1-70B) with different prompt variants were used to remove entire sections related to diagnosis, medication, and discharge (e.g., Discharge Medications, Discharge Diagnosis, Medications on Admission). Second, rule-based removal: word-matching rules were applied to identify residual diagnostic terms, which were replaced with placeholders; abbreviations of diagnoses were explicitly considered in the rules. Third, LLM validation: three open-source models (LLaMA-3.1-70B, DeepSeek-R1-70B, and Med42-70B) were employed to verify that the processed notes contained no target diagnoses. Fourth, human review: 100 notes were randomly sampled from each dataset and manually inspected to ensure processing accuracy. All reviewed notes were confirmed to be free of target diagnostic information. Notably, past medical and family histories were separately processed and highlighted within the notes to improve clarity and support subsequent data annotation.

Prompts for removing diagnosis from the notes:

SYSTEM: You are an experienced doctor who knows a lot of medical knowledge. Follow instructions EXACTLY.

USER: Extract text snippet related to the patient's chief complaints, clinical symptoms, and past medical history. The text snippet is usually before any information about "Discharge" or "Medications". Notably, do not extract any text snippet about "Discharge" or "Medications".

We used the below prompt templates to separately extract the text snippets that are used to Discharge Medications, Discharge Diagnosis, Medications on Admission, etc. These text snippets would be removed from the final notes.

Prompts for text snippet extraction from the notes:

SYSTEM: You are an experienced doctor who knows a lot of medical knowledge. Follow instructions EXACTLY.
USER: Extract text snippet related to {Admission Medications} from the note. The text snippet is usually followed by {Admission Medications} or related expressions. If the note does not include such information, you can output "none".

Prompts for generating synonyms or abbreviations of diagnosis:
SYSTEM: You are an experienced doctor who knows a lot of medical knowledge. Follow instructions EXACTLY.
USER: Please generate the commonly used synonyms or abbreviations of the diagnosis {…}. Output the results in a Python list, e.g., [‘’, ‘’, ‘’].

Supplementary Note 13

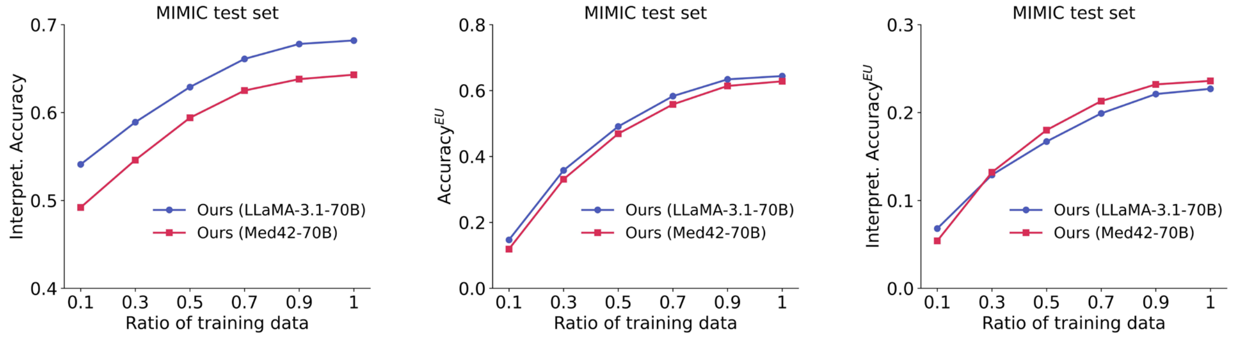

Supplementary Figure 7. Performance with varying training data size, evaluated by randomly selecting portions of the training data ranging from 10% to 90% (using metrics Interpretation Accuracy, $Accuracy^{EU}$ and Interpretation $Accuracy^{EU}$).

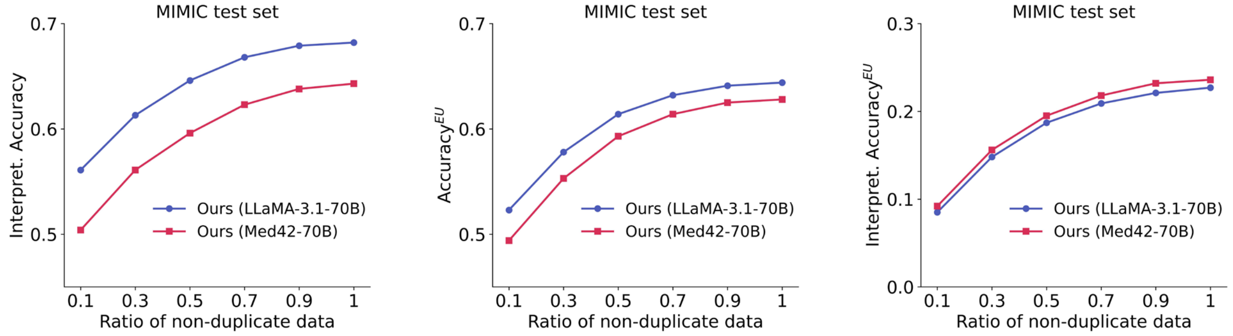

Supplementary Figure 8. Performance with varying data diversity in training data, achieved by randomly removing portions of the training samples while augmenting the remaining data to maintain a consistent training size (using metrics Interpretation Accuracy, $Accuracy^{EU}$ and Interpretation $Accuracy^{EU}$).
